# 4D formation of human embryonic forelimb musculature

**DOI:** 10.1242/dev.194746

**Published:** 2021-02-17

**Authors:** Susan Wilde, Eleanor M. Feneck, Timothy J. Mohun, Malcolm P. O. Logan

**Affiliations:** 1 Randall Centre for Cell and Molecular Biophysics, King's College London, Guy's Campus, London SE1 1UL, UK; 2 Francis Crick Institute, 1 Midland Road, London NW1 1AT, UK

**Keywords:** Human embryonic development, Congenital abnormalities, Muscle development, Muscle splitting, Upper limb

## Abstract

The size, shape and insertion sites of muscles enable them to carry out their precise functions in moving and supporting the skeleton. Although forelimb anatomy is well described, much less is known about the embryonic events that ensure individual muscles reach their mature form. A description of human forelimb muscle development is needed to understand the events that control normal muscle formation and to identify what events are disrupted in congenital abnormalities in which muscles fail to form normally. We provide a new, 4D anatomical characterisation of the developing human upper limb muscles between Carnegie stages 18 and 22 using optical projection tomography. We show that muscles develop in a progressive wave, from proximal to distal and from superficial to deep. We show that some muscle bundles undergo splitting events to form individual muscles, whereas others translocate to reach their correct position within the forelimb. Finally, we show that palmaris longus fails to form from early in development. Our study reveals the timings of, and suggests mechanisms for, crucial events that enable nascent muscle bundles to reach their mature form and position within the human forelimb.

## Abstract (precis)

**Summary:** A detailed 4D anatomical description of the human upper limb musculature through embryonic development reveals important events that enable nascent muscle bundles to form correctly.

## INTRODUCTION

The mature human limb musculature has been well described since the late 15th and early 16th centuries. Famously, Leonardo da Vinci and Vesalius provided intricate anatomical drawings that have contributed, alongside many years of cadaveric dissections and research, to produce the complete description of the upper limb musculature. Despite this, the detailed anatomy of the human muscles in the developing limb remains poorly described. A longitudinal study of human embryonic muscle formation will help us to understand the events that muscles undergo to reach their final form, origin, insertion and position, the features that together enable a muscle to carry out its functions in the mature skeletal system.

Current knowledge of vertebrate muscle formation has been dominated by experiments conducted in chicks and mice. Limb skeletal muscle precursors originate in the hypaxial domain of the somites adjacent to the limb bud, before migration into the limb bud, where they will organise into individual muscle bundles that are associated with the muscles of the mature limb (Buckingham et al., 2003; Kardon, 1998). In human development, the muscle precursor cells arrive in the upper limb bud by the seventh week of gestation (Sadler, 2012). Muscle fibres become organised into discrete domains that prefigure the muscle bundles (Besse et al., 2020; Kardon, 1998; Schroeter and Tosney, 1991a,b). Myofibres first become orientated along fixed planes. Muscle fibres with similar orientation compact together and become specific muscle bundles, a minority of which undergo a further cleavage step. These early events in primary myogenesis form the main template of muscle bundles before subsequent events expand and finely tune the mature muscle bundles (Besse et al., 2020). The tissue patterning process that creates individual muscle bundles is regulated by a population of irregular connective tissue fibroblasts, often referred to as muscle connective tissue (MCT), that surround and are embedded amongst the muscle precursors (Besse et al., 2020; Hasson et al., 2010; Kardon et al., 2003; Mathew et al., 2011). For successful muscle regeneration, satellite cells must interact with surrounding MCT for efficient regeneration (Murphy et al., 2011). Knockout studies of *Tbx5* in the mouse and misexpression in the chick demonstrate its role in early limb-bud formation and later roles in limb patterning, respectively (Hasson et al., 2010; Rallis et al., 2003). In addition, Holt–Oram syndrome, a syndrome characterised by heart and upper limb defects, including muscle dysplasia and aplasia, results from a mutation in *TBX5* (Basson et al., 1997; Li et al., 1997). When *Tbx5* is conditionally deleted from forelimb MCT cells, muscle patterning is disrupted (Besse et al., 2020), demonstrating the role of this gene, acting in MCT cells, in developing muscles with correct organisation and function.

The formation of every individual muscle is tightly regulated and, in general, occurs with a high degree of fidelity. This is reflected in the exquisite mirror symmetry of the muscles formed in the left and right sides of the body. Variations can occur, however, and muscles can be absent or there can be minor variations in muscle size and/or insertion sites, not only between individuals but also within an individual, between the right and left sides of the body. One of the most variable muscles, the forearm flexor, palmaris longus (PL), is absent in 15% of the population (Kapoor et al., 2008). Interestingly, this incidence has been shown to vary in particular ethnicities, in females and in the left versus right forearm (Nasiri et al., 2016). In tetrapod animals, the PL is involved with upper limb weight-bearing, a role that is reduced in bipedal *Homo sapiens*. The underdeveloped state or absence of the PL in humans has frequently been attributed to this difference in stance. The absence of PL is an intriguing anomaly within the population, but it is not understood whether this is caused by regression of a nascent muscle bundle or if a precursor to the mature muscle is never formed. The absence of PL has little functional significance because it makes a relatively minor contribution to forearm flexion, and two other muscles, flexor carpi radialis (FCR) and flexor carpi ulnaris (FCU), provide the bulk of flexor activity; therefore, the tendon of PL is sometimes harvested for tendon replacement surgeries. Other variations in muscles are more severe clinically and can cause patients pain and restricted movement. These comprise congenital abnormalities that are associated with muscle hypoplasia and dysplasia, which include but are not limited to Townes–Brocks syndrome, VACTERL association and Holt–Oram syndrome.

The addition and regression of muscles during the course of evolution has allowed *Homo sapiens* to adapt and develop muscles with specialisations. Specifically, the evolution of the human hand muscles has provided unique dexterous manipulation and grip, which have advanced the activities humans can carry out (Young, 2003). The unique architecture of human hand anatomy allows two grips. The precision grip enables objects to be held between the fingertips and top of the thumb, whereas the power grip allows the fingers and thumb to wrap around objects. The unique morphology of the human hand, with short metacarpals and a long thumb, contributes to its specialised function. Despite the chimpanzee having larger forearm muscles, they have significantly smaller thenar muscles, limiting the range of movement of the thumb (Ogihara et al., 2005). Specifically, adaption of the flexor pollicis longus (FPL) and the intrinsic hand muscles, the flexor pollicis brevis (FPB) and interosseous, which are missing from our ape ancestors, add strength and intricate control of the thumb, contributing to the specialised function of the human hand. Studying the development of these muscles will reveal the specific stages when they form during embryonic development and the crucial steps in their development and thereby identify when and where defects in these processes can give rise to congenital abnormalities.

A recent 3D description of the embryonic musculature system reported that all forelimb muscles are in their adult anatomical position by Carnegie stage (CS) 23 (Belle et al., 2017; Diogo et al., 2019; Warmbrunn et al., 2018). However, knowledge of earlier events and understanding of how the muscles reach their mature positions in the limb remain poor. Therefore, to probe the mechanisms used to create the great diversity in human muscle form and their intricate connections with the skeleton, we have generated a new 4D description of human embryonic forelimb muscles between CS18 and CS22 using optical projection tomography (OPT). We confirm that the forelimb muscles form in a proximal to distal direction, which is comparable to events described in the vertebrate model organisms, such as chicks and mice (Martin, 1990; Saunders, 1948; Wolpert, 1981). We show that muscle splitting and translocation of the forelimb muscles occur between CS18 and CS20. Splitting of a forming muscle bundle into two discrete units permits the formation of individual muscle heads that have specialised roles within the forelimb. Specifically, the extensor pollicis brevis (EPB) and the abductor pollicis longus (APL) begin as one muscle before splitting into two individual muscles. In addition, the extensor carpi radialis longus (ECRL) and the extensor carpi radialis brevis (ECRB) also originate as one muscle before splitting. Translocation of the flexor digitorum superficialis (FDS) from the autopod to the zeugopod is necessary for the FDS to reach its terminal position. Often a muscle that is commonly absent in the mature forelimb, we show the PL to be absent in one of our samples. This suggests that absence of the PL is the result of the bundle failing to form initially, rather than the seeding and subsequent regression of an emerging muscle bundle.

## RESULTS

### Embryonic muscle development between CS18 and CS22

**Fig. 1.**
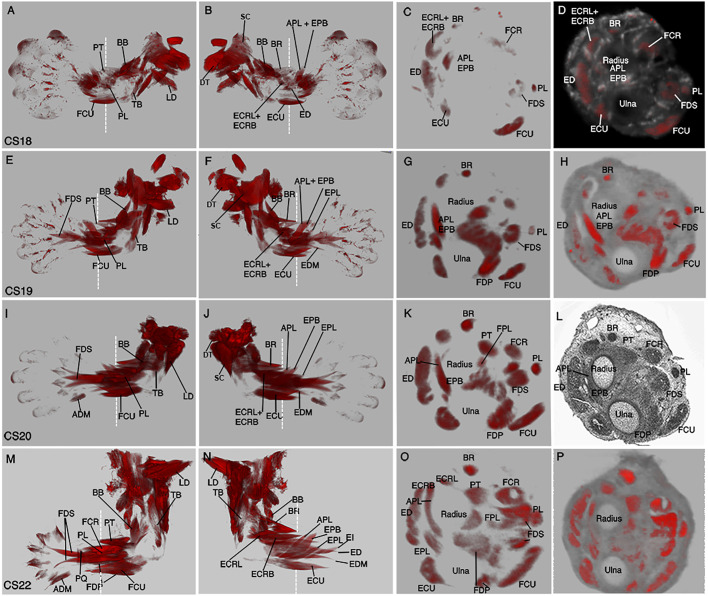
**4D** **human embryonic upper limb muscle anatomy** **between CS18 and CS22.** (A-K,M-P) Myosin-stained muscles imaged with OPT. 3D reconstructions showing the course of muscle development from CS18 to CS22. (L) High-resolution episcopic microscopy virtual section, at a comparable level to that in K, showing the histology of the CS20 embryonic limb. (A,E,I,M) Ventral/anterior compartment. (B,F,J,N) Dorsal/posterior compartment. (C,D,G,H,K,L,O,P) Distal/transverse views through the zeugopod region at the distal margin of the BR. Approximate position of virtual section is shown by the dashed white line in the ventral and dorsal views. (A) CS18: the FCU, PL and PT. (B) CS18: APL, BR, DT, ECRB, ECRL, ECU, ED, EPB and SC. (C,D) At CS18, the FDS lies deep to the PL. (E) CS19: The FDS has three extensions into the autopod. (F) The EDM and EPL are present. (I) By CS20, muscles are more mature, with the addition of ADM on the ulnar side. (J) The APL and EPB split from their radial heads and are distinguishable as individual muscle bundles from CS20. (M-P) CS22: the ED, EI and PQ. (M,N) The stylopod muscles BB, LD and TB. At CS22, the dorsal muscles have been removed from the ventral view, and in the dorsal view the ventral muscles have been extracted. ADM, abductor digiti minimi; APL, abductor pollicis longus; BB, biceps brachii; BR, brachioradialis; DT, deltoid; ECRB, extensor carpi radialis brevis; ECRL, extensor carpi radialis longus; ECU, extensor carpi ulnaris; ED, extensor digitorum; EDM, extensor digiti minimi; EI, extensor indicis; EPB, extensor pollicis brevis; EPL, extensor pollicis longus; FCU, flexor carpi ulnaris; FDS, flexor digitorum superficialis; LD, latissimus dorsi; PL, palmaris longus; PQ, pronator quadratus; PT, pronator teres; SC, subscapularis; TB, triceps brachii.

At CS17, we are unable to detect any distinct muscle bundles using the MY-32 myosin antibody. At CS18, a subset of muscle bundles is morphologically discernible, and at the later stages analysed (CS19-CS22) there is a generalised progression of muscle differentiation and maturation from proximal (shoulder/arm) to distal (hand) (Fig. 1). In the stylopod, at all ages analysed the biceps brachii (BB), triceps brachii (TB), latissimus dorsi (LD), subscapularis (SC), deltoid (DT) and brachioradialis (BR) muscles are distinguishable. At CS18, within the forearm flexor compartment the muscles of the most superficial layers, the pronator teres (PT), FCU, FCR, FDS and the PL are observed. In the forearm extensor compartment again the most superficial layer muscles, the extensor carpi ulnaris (ECU), ECRB, ECRL and extensor digitorum (ED) can be seen. (Fig. 1A-D; Movies 1 and 2). As development proceeds (CS19-CS20), muscles of the deep layers become visible; for example, in the flexor compartment the flexor digitorum profundus (FDP) and PL and in the extensor compartment the APL and extensor pollicis longus (EPL) (Fig. 1E-L; Movies 3-6). Therefore, there is a general trend that muscles of the most superficial layers are formed in advance of muscles of deeper layers.

Coincident with new muscles developing in deep layers, previously underdeveloped muscles of the more superficial layers mature their anatomy and position within the upper limb. The FDS at CS19-CS20 is more prominent, with three extensions directed towards the autopod (Fig. 1E,I). At CS19, the EPL and extensor digiti minimi (EDM) are identified for the first time (Fig. 1F). At CS20, all the muscles mentioned previously have now enlarged, and the abductor pollicis longus (APL) and EPB are identified as individual muscles (Fig. 1I-L). Muscles previously established enlarge further by CS22, and muscle differentiation forms additional separated muscles (Fig. 1M-P; Movies 7 and 8). At CS22, the pronator quadratus (PQ) on the distal zeugopod spans across the radial and ulnar regions (Fig. 1M). On the dorsal side, the extensor indicis (EI) and ED are distinguishable amongst the extensor forearm muscles lengthening towards the autopod (Fig. 1N). Our data demonstrate that by CS22, most upper limb muscles have developed the primary aspects of their mature form and position within the limb by week 8 of embryonic development.

**Fig. 2.**
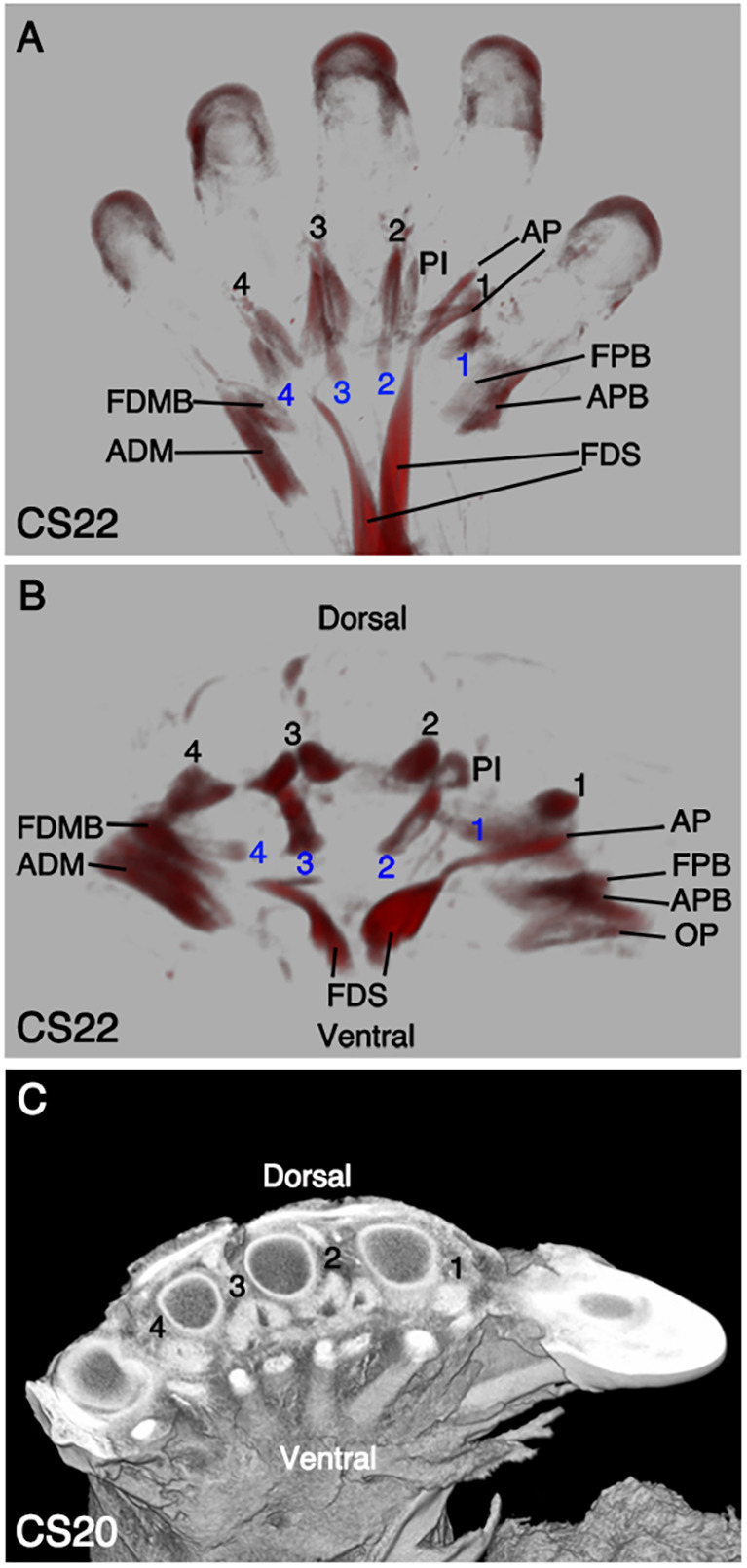
**Anatomy of the autopod muscles.** (A,B) Myosin-stained muscles imaged with OPT. (A) Ventral view at CS22 shows the thenar, hypothenar, interosseous and lumbrical muscles. The hypothenar eminence includes the ADM and FDMB on the ulnar side. The FPB, AP and APB make up the thenar eminence on the radial side. The FDS within the autopod has two extensions, with its main muscle belly located proximally. The intrinsic PI on the ventral surface sit between the metacarpal bones. (B) Distal-transverse view shows the dorsal and ventral surfaces of the autopod. The AP, FPB, APB and OP are positioned from medial (ulnar) to lateral (radial), respectively. There are four dorsal interosseous muscles (1-4, black) and lumbricals (1-4, blue). (C) High-resolution episcopic microscopy of the CS20 autopod shows the relative positions of the bones, tendons and muscles. ADM, abductor digiti minimi; AP, abductor pollicis; APB, adductor pollicis brevis; APL, abductor pollicis longus; BB, biceps brachii; BR, brachioradialis; DT, deltoid; ECRB, extensor carpi radialis brevis; FDMB, flexor digiti minimi brevis; FDS, flexor digitorum superficialis; FPB, flexor pollicis brevis; OP, opponens pollicis; PI, palmar interossei.

The intrinsic muscles of the autopod are first detected in the ventral compartment at CS20, with the identification of abductor digiti minimi (ADM) (Fig. 1I). At CS22, the first palmar interosseous muscle is visible (Fig. 2A). In some individuals, four interosseous muscles can be present instead of three, with the first palmar interosseous thought to be rudimentary. If this occurs, the identified muscle would be the second palmar interosseous muscle. On the dorsal surface, four dorsal interossei along with the previously described palmar interossei occupy the space between the metacarpal bones (Fig. 2B). Four lumbrical muscles that usually lie deep to the palmar fascia are also present (Fig. 2B). All hypothenar and thenar muscles are distinguishable in the embryonic human hand at CS22 (Fig. 2). On the ulnar side, the ADM and flexor digiti minimi brevis (FDMB). On the radial side, the opponens pollicis (OP), APB, FPB and adductor pollicis (AP) form the thenar eminence (Fig. 2). The intricate development of the thenar muscles is essential to control the wide range of movements of the thumb, which adds dexterity to the hand.

Detailed histology of the embryonic forelimb at stage CS20 was revealed with high-resolution episcopic microscopy (Fig. 1L; Fig. 2C; Movie 9). The 3D reconstruction with this technique displays the relationship between the bones, muscles, tendons and surrounding connective tissue. We show the muscles to be organised tightly around the radius and ulna at CS20, and they are already compartmentalised into their individual muscles and muscle groups by the surrounding connective tissue (Fig. 1L). In the autopod, the ventral tendons of the FDS are clearly inserted by CS20 between the three FDS muscle bundles (Fig. 1I), which is important for the FDS to carry out the process of translocation for the FDS to reach its terminal position within the zeugopod (Fig. 2C).

### Muscle splitting forms individual muscles

**Fig. 3.**
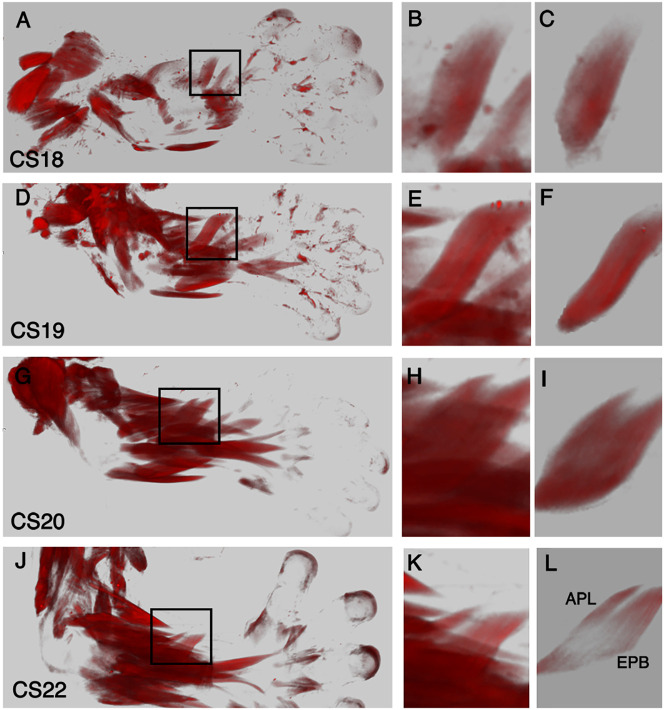
**Formation of the** **APL and the EPB.** Myosin-stained muscles imaged with OPT. 3D reconstructions of specimens between CS18 and CS22 in dorsal view. (B,E,H,K) The APL and EPB are shown at a higher magnification. (C,F,I,L) Virtual dissection reveals the APL and EPB without surrounding muscles. (A,D,G,J) The muscle bellies of APL and EPB are within the black box. (A-F) From CS18 to CS19, a single muscle bundle exists in the ventral zeugopod. (C,F,I,L) Virtual dissections of the datasets reveal the APL and EPB becoming separate bundles through ‘splitting’/cleavage of a ‘parental’ bundle. (G-I) By CS20, the muscle is split from the distal tip. (J-L) At CS22, the APL and EPB continue to separate towards the proximal origin. APL, abductor pollicis longus; EPB, extensor pollicis brevis.

To understand the events that form individual muscles, several of those in the zeugopod were analysed in greater depth over the course of development from CS18 to CS22. The APL and EPB are described to form through a process of muscle ‘splitting’, which is the formation of two distinct muscle bundles from a single parental bundle (Fig. 3). At CS18 and CS19, a single muscle bundle is present in the dorsal zeugopod at the anatomical position where the APL and EPB are positioned within the adult (Fig. 3A-F). This bundle begins to split from its distal end by CS20, and this progresses from distal to proximal, creating two individual muscle bundles by CS23 (Fig. 3G-L). By tracing the muscles, we find that the splitting continues at CS22 in an ulnar direction but does not split completely through the ulnar root. Both muscles are deep extensor muscles of the forearm that share a similar origin from the radial surface and interosseous membrane. The mature APL has an additional origin on the ulna, which is evident at CS22 with the APL extending towards the ulna (Fig. 3L). The ECRL and ECRB originate as one muscle, but by CS22 they have split into two distinct muscles (Fig. 1J,N).

### Translocation of muscles to their terminal position

**Fig. 4.**
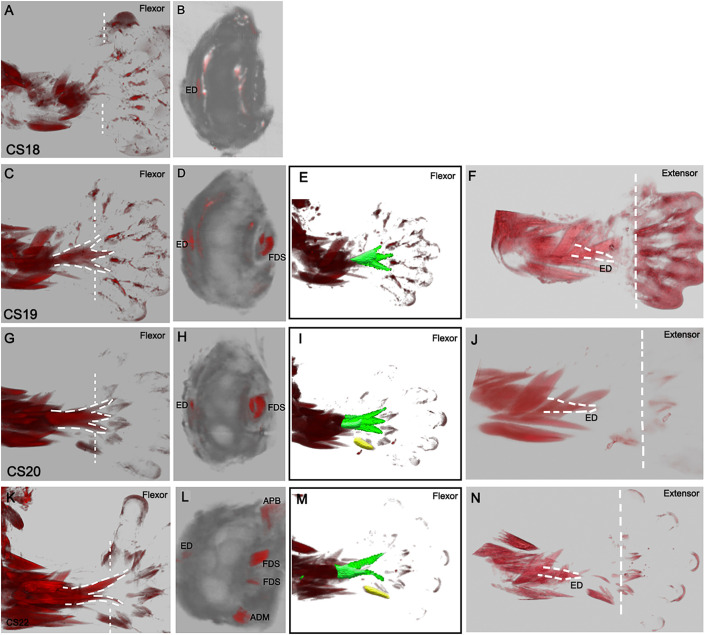
**Proximal translocation of the** **FDS and the ED.** Myosin-stained muscles imaged with OPT. 3D reconstructions of specimens at CS18 (A,B), CS19 (C-F), CS20 (G-J) and CS22 (K-N). (C) The FDS is visible with three bellies at CS19. (C,G,K) The FDS in the ventral autopod translocates proximally into the zeugopod between CS19 and CS22. (K) At CS22, the FDS has two bellies that extend into the autopod. (B,D,H,L) Transverse, virtual sections at the autopod and zeugopod border showing the positions of FDS and ED muscles between CS19 and CS22. (E,I,M) The FDS (green) and the ADM (yellow) are segmented to visualise the translocation. The FDS moves proximally with age, in comparison to the ADM, which remains distally located at CS22. (F,J,N) The ED translocates CS19 and CS20. White lines denote the approximate equivalent location between ages. ADM, abductor digiti minimi; ED, extensor digitorum; FDS, flexor digitorum superficialis.

We describe the translocation of muscles from a distal to proximal position to reach their mature location within the upper limb. The FDS is initially seen at CS19, with three distal muscle extensions in the ventral autopod (Fig. 4C,D). At CS20, the FDS matures, with an enhanced proximal muscle belly projecting towards the distal zeugopod. By CS22, the proximal muscle bellies translocate into the zeugopod. The FDS at CS22 appears with two distal muscle extensions, similar to the humeroulnar and radial heads that are found in the mature forelimb, with subsequent tendon attachments in the hand. To characterise the course of FDS translocation further, the FDS was segmented alongside the initially neighbouring intrinsic hand muscle, ADM, and in addition, transverse cross-sections are shown at the embryonic wrist (Fig. 4). At CS20, the FDS is lying adjacent to the ADM. However, by CS22 the FDS has translocated proximally, whilst the ADM remains distal (Fig. 4E,I,M). The FDS between CS19 and CS20 is seen as a single object in cross-section. By CS22, two individual muscles are identified that are not located within the same plane, which also demonstrates that the FDS continues to translocate proximally. The FDS can also be seen with two muscle components directed towards digits two and four in the autopod (Fig. 2A). In the mature forearm, the FDS splits into four tendons before reaching the wrist in a more proximal location than witnessed in the embryo, and the tendons then attach to the second to fifth middle phalanges to allow the FDS to flex digits two to five.

In the transverse cross-section, the ED is identified in the dorsal region of the embryonic autopod-zeugopod border (Fig. 4). In the adult, the forearm extensors are more proximally located compared with their distal location in the embryo. This suggests that the extensors in the dorsal autopod translocate proximally to reach their terminal position in the adult forelimb. The ED at CS22 has two muscle components, similar to FDS at this stage (Fig. 4L). In the posterior view, the ED translocates proximally between CS19 and CS20, moving away from the lumbricals (Fig. 4F,J,N). We show that both the FDS and the ED translocate proximally between CS18 and CS22, which displays homology in the development between the flexor and extensor muscles.

### The absence of PL

**Fig. 5.**
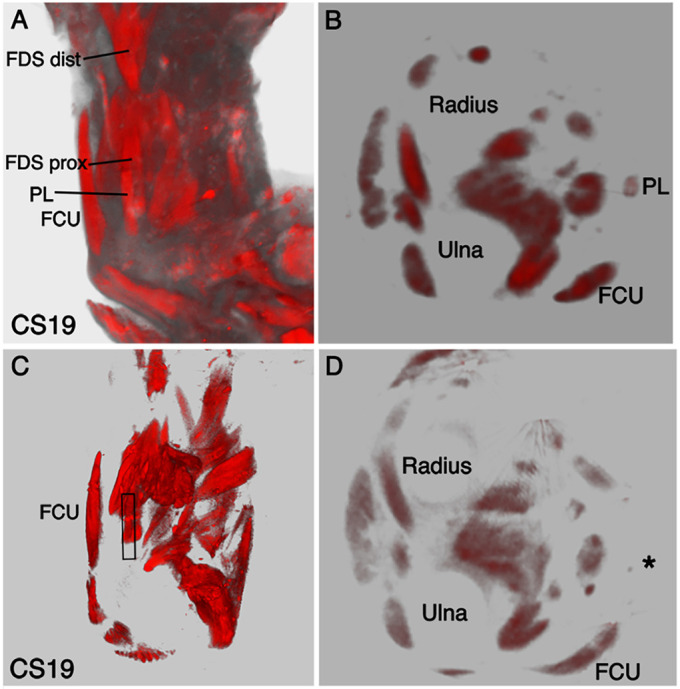
**Absence of the** **PL** **at CS19.** Myosin-stained muscles imaged with OPT. (A) At CS19, the PL is superior to the FDS and medial to the FCU in the ventral view. (B) In a transverse virtual section at the distal edge of the brachioradialis, the PL is a superficial cylindrical muscle. (C,D) The PL is absent in one CS19 sample. (C) At the elbow joint, the black box represents the location where the PL should be present, with the FCU positioned lateral to the PL. (D) In a transverse section of the same sample as shown in C, the PL is not apparent, with the expected position represented by a black asterisk. FCU, flexor carpi ulnaris; FDS, flexor digitorum superficialis; PL, palmaris longus.

The forming PL can be identified at CS18 and is traced through development until the oldest age analysed, CS22 (Fig. 1). In one sample out of seven limbs scanned at CS19, the PL could not be identified in the 3D reconstruction (Fig. 5). In the limbs where the PL is identified at CS19, the muscle has a thin cylindrical morphology that arises at the proximal end of the zeugopod, close to the FCU and superficial to the FDS (Fig. 5A,B). The PL shows a similar anatomy and course by CS19 compared with the adult forelimb, where it originates from the medial epicondyle of the humerus with a short muscle belly in the proximal forearm before its long tendon extends down the forearm. The PL is the most variable muscle in the human body, and our results demonstrate that the PL can be absent from CS19 (Fig. 5C,D). In this sample, it is possible that the PL has failed to form rather than regressing after formation.

## DISCUSSION

We describe a 4D dataset of human upper limb muscle development between CS18 and CS22. These data provide new insights into the normal development of human upper limb musculature. Understanding the normal development provides clues to the underlying causes of hypoplastic or dysplastic muscles and tendons associated with congenital abnormalities. Between CS18 and CS22, individual muscles are initially formed and mature *in situ* with, for some muscles, the addition of translocation and splitting that fine-tune their form and final position. We observe the general trend that proximal muscles form in advance of the more distal muscles and, at least within the zeugopod region, that the most superficial muscles mature in advance of the muscles of deeper layers. Muscles of the deep layers have insertion on the skeleton at more distal locations than muscles of more superficial layers. It is possible, therefore, that the maturation of muscles is correlated with the time at which the distal tendon of each muscle is able to insert at its appropriate site on the forming skeleton. Induction of distal limb tendons is reliant on signals from cartilage, and the proximal tendons rely on interactions with muscle (Huang et al., 2015); therefore, the cues that signal to the distal and proximal tendons are divergent. The maturation of deeper muscles that insert their tendons distally could be delayed as a consequence of the later maturation of the most distal cartilaginous elements.

We show that the APL and EPB arise from a common, single muscle bundle. The separation or ‘splitting’ process that creates these two muscles is initiated by CS20 from the distal end of the muscle and progresses in a distal to proximal direction. We observed a similar distal to proximal ‘splitting’ process in the mouse, whereby the extensor carpi radialis splits into the distinct ECRL and ECRB (Besse et al., 2020), as we show here, in the human. At CS22, the APL and EPB still share a common origin and, as development progresses, we would predict splitting to continue as the muscles reach their terminal position. In the mouse, a single extensor pollicis muscle is found in the equivalent position to the APL and EPB in the human, but the splitting event does not occur (Delaurier et al., 2008). In order to acquire the APL and EPB in the human, therefore, a radical change in the splitting events must have occurred. Although rare, the APL and EPB can remain fused in humans with abnormal tendon insertions (Dhuria et al., 2012). In addition, congenital clasped thumb (the thumb is fixed into the palm of the hand at the metacarpophalangeal joint) can present if there is an absence of either one of these muscles (Ghani et al., 2007; Jochims, 1983). A failure of the splitting event we describe to form the APL and the EPB between CS18 and CS22 could explain the apparent ‘fusion’ of these two muscles and associated defects in the range of motion of the thumb.

The exact mechanisms that regulate muscle splitting remain unclear. Ultimately, understanding these events would allow us to recognise how their failure could lead to muscle dysmorphologies seen in congenital abnormalities. Studies in the developing chick limb have reported that the dorsal and ventral muscles separate via regulation from blood vessels (Tozer et al., 2007). In the mouse, conditional deletion of *Tbx5* in MCT cells disrupts muscle bundle ‘splitting’. *Tbx5* acts in connective tissue fibroblast cells to modulate the ECM milieu, which could have a role in the splitting process (Besse et al., 2020). APL and EPB have adjacent origins but distinct insertions at the base of the first metacarpal and the dorsal aspect of the proximal phalanx of the thumb, respectively. Although it is unclear whether external forces are acting to drive the splitting process we describe, it is possible that maturation of the physically distinct insertion sites enables the single bundle to be ‘prised apart’ in the distal to proximal pattern that we observe. Muscle contraction is essential in the development of tendon to bone insertion sites, with an absence of muscle force affecting bone shape and size (Mikic et al., 2000; Ogawa et al., 1999). In a Desmin mutant mouse, in which muscle contraction force is weakened, a loss of muscle splitting is observed (Diermeier et al., 2017). Absent or reduced muscle contraction during human embryonic development could therefore contribute to failed muscle splitting, leading to aberrant muscles remaining in the adult.

We describe the translocation of the FDS, which forms initially in the autopod and subsequently moves to its final position within the anterior compartment of the zeugopod. This observation is equivalent to the translocation of the FDS that has been described in the mouse and is dependent on muscle contraction and tendon attachment (Huang et al., 2013). Long tendons anchor and then elongate and lengthen within the zeugopod, via the recruitment of mesenchymal cells (Huang et al., 2019). A failure of this mechanism could contribute to aberrant muscle masses within the hand. In humans, muscle can remain attached to the mature FDS tendons within the hand, with some case reports describing ectopic bellies remaining in the hand and wrist (Elliot et al., 1999). Clinically, muscle remnants in these locations can cause nerve entrapment and pain in patients (Still and Kleinert, 1973). Specifically, aberrant muscle distal to the carpal tunnel can compress the median nerve during wrist flexion and extension (Javed and Woodruff, 2014). A failure of the muscles to contract and translocate or a delay/failure of tendons to attach could result in the anomalies described.

Additionally, we describe, for the first time, translocation of the human dorsal forelimb extensor muscles from the autopod into the zeugopod. An equivalent translocation of extensor muscles has not been described in the mouse. Remnants of the extensor digitorum brevis have been described in the hand (Ranade et al., 2008; Still and Kleinert, 1973), that suggesting a failure of translocation into the forearm is consistent with an equivalent translocation event occurring normally within the extensor muscle compartment. We show that the dorsal and ventral muscle bundles develop following similar dynamics to reach their terminal position within the mature forelimb. In amphibians and reptiles, the flexor muscles are located within the hand (Abdala and Diogo, 2010; Wesser et al., 1969). We find that in human embryonic development, the flexor muscles initially form in the autopod before translocating proximally into the forearm, starting development in a position similar to where the muscles are located in mature evolutionary ancestors. A recent study of human fetal upper limb musculature reported the presence of an ectopic muscle in the dorsal hand. That study identified this muscle as the ‘dorsometacarpalis’ and suggested that it represents a transient, atavistic muscle that subsequently regresses (Diogo et al., 2019). We find no evidence of muscles in the posterior compartment of the hand in any of the specimens we have analysed between CS18 and CS22, nor do we find any muscle bundles that are not present in the adult. This is consistent with literature from the early 20th century describing the intrinsic hand muscles in human embryonic development (Grafenberg 1905, Lewis 1910). Grafenberg states that short extensors of the hand are never present in human ontogeny (Grafenberg 1905). Lewis describes muscle bundles that form the interossei within the palmar surface of the hand that migrate towards the dorsal surface, between the metacarpals, while remaining in the anterior compartment. In contrast, comparative anatomy and human embryology studies (reviewed by Cihák, 1972) that follow a 'recapitulation rule' (ascribe to the ‘ontogeny recapitulates phylogeny' concept) have reported the presence of interossei dorsalis accessorii (equivalent to the dorsometacarpalis) in the posterior compartment of the autopod. The reported innervation of these muscles by the ulnar nerve could be consistent with an origin in palmar muscle masses followed by dorsal intermetacarpal translocation. Based on our results, we argue that the presence of an atavistic ‘dorsometacarpalis’/interossei dorsalis accessorii muscle(s) is not part of normal human embryonic muscle development. While the older literature is extensive, the tools used to stain and image tissues were limited, making definitive identification of structures from tissues sections problematic. The application of contemporary immunohistochemical stains and microscopy methods offer potential for further study. Additional imaging of embryonic samples across a broader range of stages and with additional tissue markers, will help to resolve this discrepancy.

The PL is a distinctive cylindrical muscle with a long tendon that expands down the forearm to insert into the palmar aponeurosis and flexor retinaculum in the hand. The PL has a role in loadbearing (tetrapod) mammals and, with its diminished role in bipedal humans, it has regressed. We show that the PL can be absent from CS19 of embryonic development, suggesting that in individuals where PL is completely absent, the muscle fails to form rather than regressing after being present. There are cases where the PL forms abnormally or leaves ectopic muscle constituents within the wrist. In these individuals, it is possible that the muscle forms abnormally, which leads to the muscle degenerating or gives rise to variations in its course, attachment, origin or position within the mature human forelimb (Ioannis et al., 2015). The absence of the PL was seen in only one of three samples, demonstrating variability between the human samples analysed. Human embryonic material is rare, necessitating the low numbers of samples analysed. Variability in morphogenetic processes is expected in the human population, and a larger number of samples would allow us to understand these variations in greater detail. However, in our longitudinal study, we analyse multiple samples across a defined developmental time window, which allows us to detect reproducible and progressive maturation of muscle morphology.

**Fig. 6.**
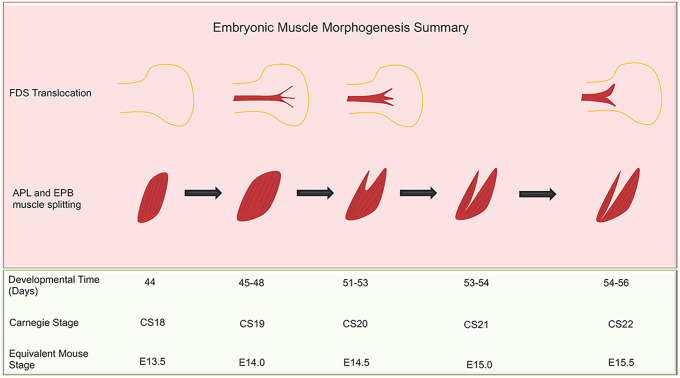
**Summary of embryonic muscle morphogenesis.** The human CS and mouse stage equivalent are represented alongside the human developmental time in days. The translocation of the FDS and the splitting of the APL and EPB are shown over the stages at which these events occur in human development. APL, abductor pollicis longus; EPB, extensor pollicis brevis; FDS, flexor digitorum superficialis.

The evolutionary changes of the intrinsic hand muscles have afforded humans sophisticated manual dexterity. We show the intrinsic hand muscles develop initially at CS20 and are individually distinct by CS22. The adaptations in the development of our intrinsic hand muscles, with shorter metacarpals and a longer thumb, have granted us dextrous abilities unavailable to other species. The extrinsic FPL and EPB provide the thumb with its large range of motion, in particular opposition, flexion and extension. Our data describe some of the unique events and features that sculp the anatomy of human hand and upper limb musculature. The muscle morphological events corresponding to the specific human embryonic time and CS alongside the equivalent mouse stage are summarised (Fig. 6). Furthermore, any interruption to the events we describe can result in predictable muscle dysplasia, i.e. hypoplasia associated with congenital upper limb defects.

## MATERIALS AND METHODS

### Collection of human embryonic limbs

Human embryonic limbs staged between CS18 and CS22 were obtained from the Medical Research Council–Wellcome Trust Human Developmental Biology Resource (HDBR), fixed in 4% paraformaldehyde (PFA) and stored at 4°C before arrival. The HDBR tissue bank is regulated by the Human Tissue Authority (HTA) and operates under their codes of practice. The upper limb of embryonic samples CS18 (one), CS19 (three), CS20 (one) and CS22 (two) were processed. All tissue preparation was carried out in accordance with HTA guidelines. On arrival, the limbs were hydrated in PBS and skinned in PBS with 0.1% Triton X-100 for 30 min.

### Myosin labelling

Samples were blocked in PBS with 1% bovine serum albumin, 0.15% glycine and 0.1% Triton X-100 at 4°C for 1.5-4 h. Limbs were stained with MY-32 mouse monoclonal anti-skeletal myosin (fast) antibody (Sigma, M4276) directly conjugated to fluorophores using Zenon Texas Red-X Mouse IgG1 Labelling kit Z25045 or labelled with Zenon Alexa Fluor 594 Mouse IgG1 Labelling Kit Z25007 (Molecular Probes) according to the manufacturer's protocol. Conjugated antibody was used at a dilution of 1:800 and incubated with specimens at 4°C for 48 h. Samples were then washed seven times in PBS with 0.1% Tween-20 (PBT) at 4°C for 5 h and, finally, overnight at 4°C. Limbs were postfixed in 4% PFA in PBT for 30 min, washed in PBT and stored at 4°C in PBS.

### Preparation of limbs for OPT

Limbs were embedded in 1% low-melting-point agarose (Sigma, A9414), following steps 3-12 of a previously described protocol (Quintana and Sharpe, 2011). Agarose blocks contained one limb, dehydrated in 96% ethanol for 19 h, with two changes of solution to preserve the fluorescence (Oldham et al., 2008; Sakhalkar et al., 2007). The 96% ethanol was replaced with a clearing agent, BABB [1:2 mixture of benzyl alcohol 402834 (Sigma) and colourless benzyl benzoate 12392-F from Fluka (Sigma)], changed three times over 24 h. A drop of Loctite Super Glue Precision Max was placed on a cylindrical stainless-steel mount 10 mm in width, and the base of the agarose block was stuck to it and left to dry for 15 min in the dark before OPT scanning (Sharpe et al., 2002; Quintana and Sharpe, 2011).

### Image collection

OPT images were obtained using a Leica MZ FLIII microscope body and ultraviolet light source fitted with a green fluorescent protein 1 (GFP1) filter used to capture autofluorescence of the whole limb and a rhodamine filter set used to image the Texas Red-labelled muscles. The specimen was imaged to retrieve 3D datasets as described previously (DeLaurier et al., 2006). Digital scans of limbs were reconstructed into virtual transverse sections. ImageJ was used to colour the *z*-stack of up to 670 sections, to merge scans from both filters and to carry out segmentation of the individual muscles. 3D rendering of the limbs was carried out using OsiriX (Pixmeo). Datasets were converted into TIFF images or 3D QuickTime movies.

### High-resolution episcopic microscopy

Samples fixed in 4% PFA and transferred into PBS were embedded within a resin block and prepared for high-resolution episcopic microscopy imaging according to a previously described protocol (Weninger et al., 2006).

## Supplementary Material

Supplementary information
