# Supplementary material for: 4D formation of human embryonic forelimb musculature
Source: Development. 2021 Feb 17;148(4):dev194746. doi: 10.1242/dev.194746 (PMC7904005; doi:10.1242/dev.194746)
Supplement: Supplementary information [file develop-148-194746-s1.pdf]

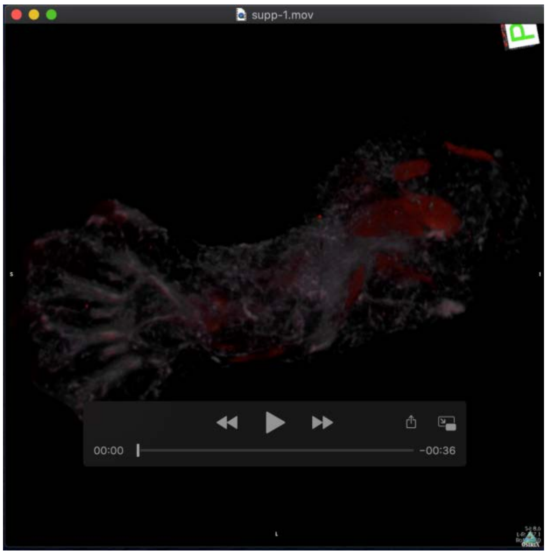

Movie 1. 3D human upper limb muscle anatomy at CS18. Myosin-stained upper limb muscles imaged by optical projection tomography rotated to show the muscles in the autopod, zeugopod and stylopod in anterior/ventral and posterior/dorsal view.

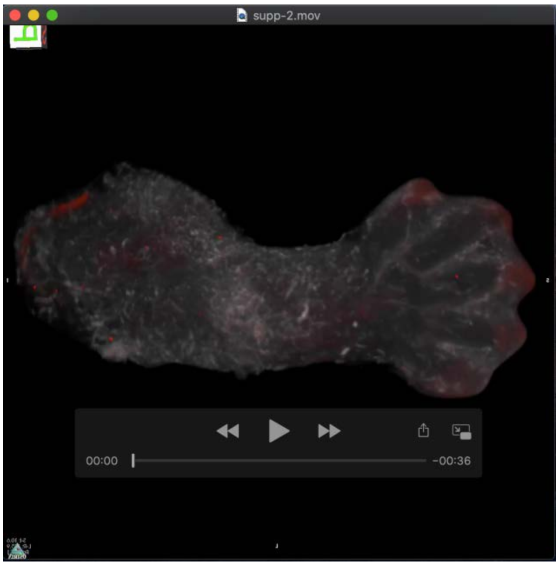

Movie 2. 3D human upper limb muscle anatomy at CS18. Myosin-stained upper limb muscles imaged by optical projection tomography. The model is subsequently cropped to show the upper limb muscles in cross-section.

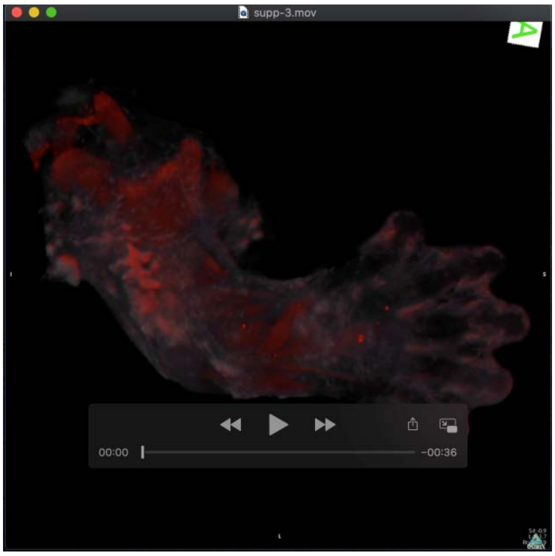

Movie 3. 3D human upper limb muscle anatomy at CS19. Myosin-stained upper limb muscles imaged by optical projection tomography rotated to show the muscles in the autopod, zeugopod and stylopod in anterior/ventral and posterior/dorsal view.

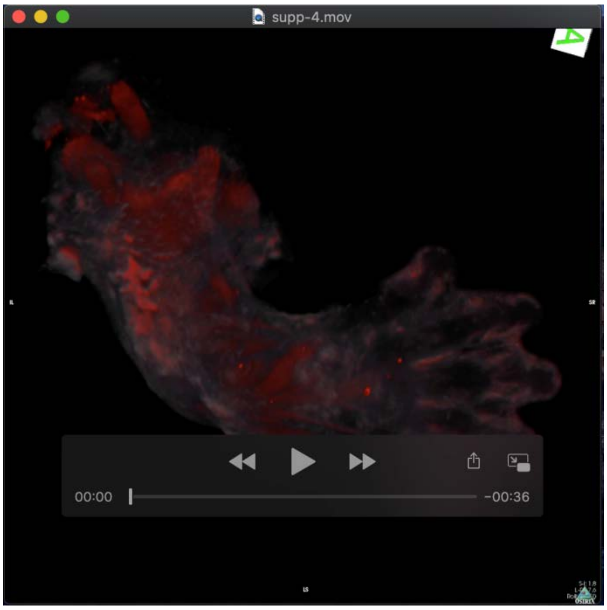

Movie 4. 3D human upper limb muscle anatomy at CS19. Myosin-stained upper limb muscles imaged by optical projection tomography. The upper limb muscles are viewed in cross-section.

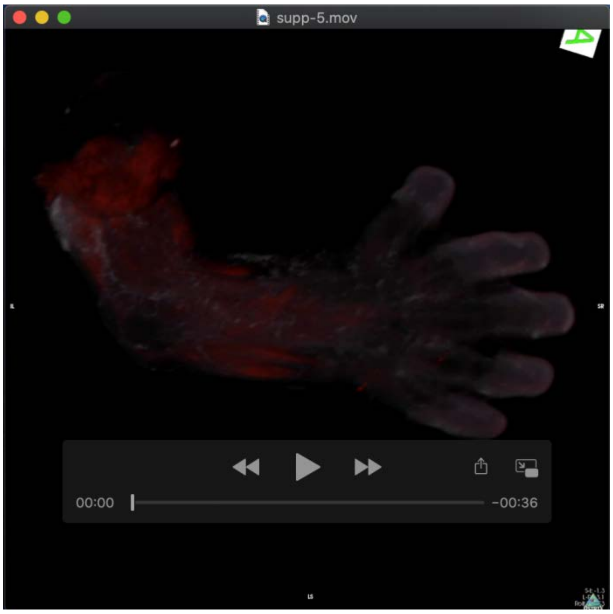

Movie 5. 3D human upper limb muscle anatomy at CS20. Myosin-stained upper limb muscles imaged by optical projection tomography and rotated to show the muscles in the autopod, zeugopod and stylopod in anterior/ventral and posterior/dorsal view.

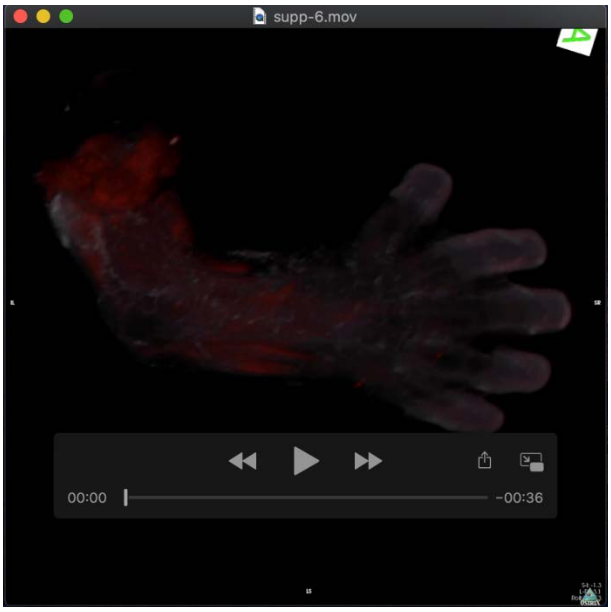

Movie 6. 3D human upper limb muscle anatomy at CS20. Myosin-stained upper limb muscles imaged by optical projection tomography are viewed in cross-section.

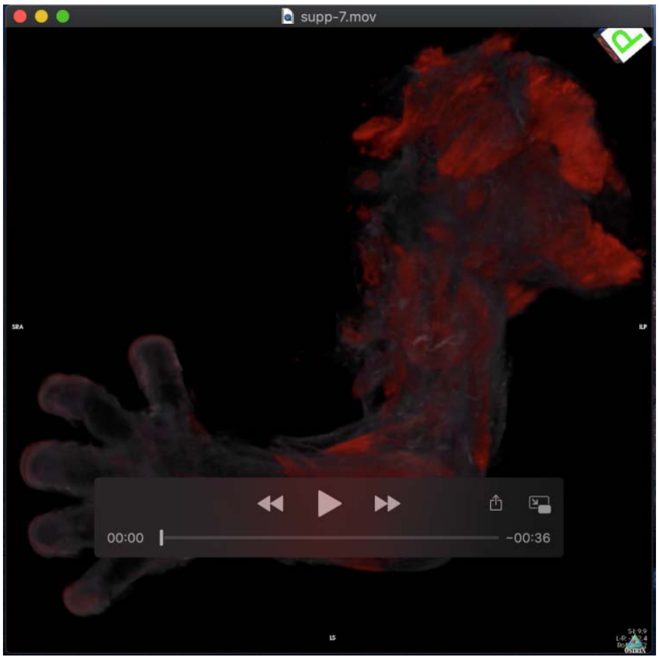

Movie 7. 3D model of the upper limb muscle anatomy at CS22. Myosin-stained upper limb muscles imaged by optical projection tomography. The reconstruction is rotated to show the muscles in anterior/ventral and posterior/dorsal view.

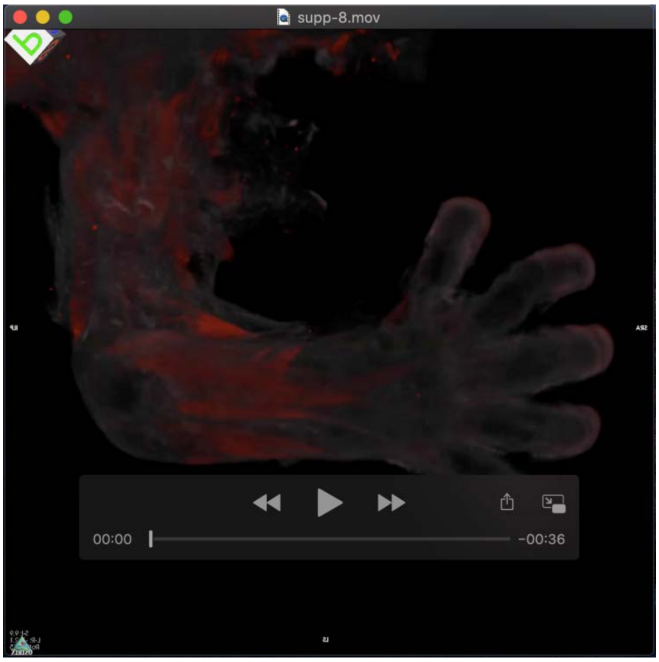

Movie 8. 3D model of the upper limb muscle anatomy at CS22. Myosin-stained upper limb muscles imaged by optical projection tomography. Reconstructed 3D models are viewed in cross-section.

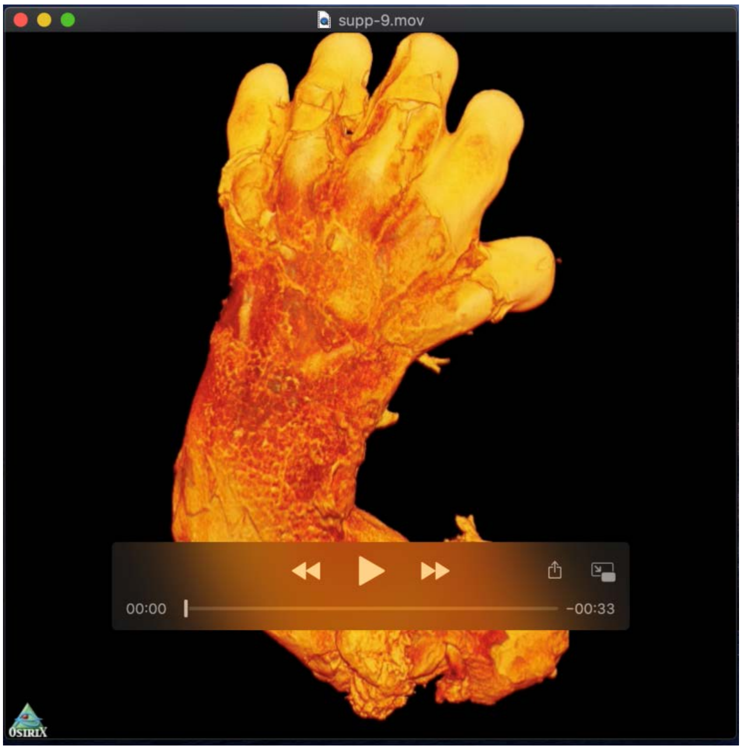

Movie 9. 3D human upper limb muscle anatomy at CS20. High resolution-episcopic microscopy (HREM) imaged upper limb viewed as a volume render. HREM imaging provides a high-resolution 3D model showing the relationship between the muscles, bone and tendons. The reconstruction is rotated and rendered to view the anterior/ventral, dorsal/posterior compartments and a cross-sectional view of the upper limb.

Development • Supplementary information
